# A systematic review of the impact of center volume in dialysis

**DOI:** 10.1186/s13104-015-1785-5

**Published:** 2015-12-22

**Authors:** Dawid Pieper, Tim Mathes, Mark Roger Marshall

**Affiliations:** Institute for Research in Operative Medicine, Witten/Herdecke University, Ostmerheimer Str. 200, Building 38, 51109 Cologne, Germany; Faculty of Medical and Health Sciences, University of Auckland, Auckland, New Zealand; Department of Renal Medicine, Counties Manukau, Health, Auckland, New Zealand; Baxter Healthcare (Asia Pacific), Shanghai, People’s Republic of China

**Keywords:** Dialysis, Hospitals, high volume, Kidney failure, Outcome assessment, Review

## Abstract

**Background:**

A significant relationship exists between the volume of surgical procedures that a given center performs and subsequent outcomes. It seems plausible that such a volume–outcome relationship is also present in dialysis.

**Methods:**

MEDLINE and EMBASE were searched in November 2014 for non-experimental studies evaluating the association between center volume and patient outcomes [mortality, morbidity, peritonitis, switch to hemodialysis (HD) or any other treatment], without language restrictions or other limits. Selection of relevant studies, data extraction and critical appraisal were performed by two independent reviewers. We did not perform meta-analysis due to clinical and methodological heterogeneity (e.g. different volume categories).

**Results:**

16 studies met out inclusion criteria. Most studies were performed in the US. The study quality ranged from fair to good. Only few items were judged to have a high risk of bias, while many items were judged to have an unclear risk of bias due to insufficient reporting. All 10 studies that analyzed peritoneal dialysis (PD) technique survival by modeling switch to HD or any other treatment as an outcome showed a statistical significant effect. The relative effect measures ranged from 0.25 to 0.94 (median 0.73) in favor of high volume centers. All nine studies indicated a lower mortality for PD in high volume centers, but only study was statistical significant.

**Conclusions:**

This systematic review supports a volume–outcome relationship in peritoneal dialysis with respect to switch to HD or any other treatment. An effect on mortality is probably present in HD. Further research is needed to identify and understand the associations of center volume that are causally related to patient benefit.

**Electronic supplementary material:**

The online version of this article (doi:10.1186/s13104-015-1785-5) contains supplementary material, which is available to authorized users.

## Background

Previous systematic reviews (SR) have shown the presence of a significant relationship between the volume of surgical procedures that a given center performs and subsequent outcomes [1–6]. This so-called volume–outcome relationship is reported to be stronger in high risk, low volume procedures [7–10]. The volume–outcome relationship has also been found outside of surgery [11, 12]. Two hypotheses exist for this relationship. On the one hand, a higher caseload and experience may result in more effective skills (“practice makes perfect”). On the other, providers with better outcomes might receive more referrals thereby increasing their volume (“selective referral”) [13, 14].

Outcomes in dialysis are very heterogeneous in international research [15–17]. Such differences even remain after adjusting for measured patient characteristics [18]. Acknowledging residual confounding as an ever-present issue, center-related attributes are likely to be the main contributing factor. It is plausible that volume–outcome relationships are causal or intermediate variables on the pathway between center effect and patient outcomes (e.g. mortality risk).

To the best of our knowledge, there is no reported SR on the volume–outcome relationship in dialysis. We also did not find any ongoing SR by checking registries (Cochrane library and PROSPERO). The aim of this SR is to examine the effects of center volume, specialization and regionalization on the outcomes in dialysis.

## Methods

We performed a systematic literature search to identify all relevant publications on the relationship between center volume and patient outcomes in dialysis. Medline (via PubMed) and Embase (via Embase) were searched from inception to November 2014 (see Additional file 1: Appendix S1 for search strategies). Reference lists of retrieved articles were inspected to identify additional articles that could have been missed by our search strategy. No language restrictions or other limits were applied.

For consideration in this SR the following inclusion criteria were applied to each publication: the subject of the study was dialysis [either peritoneal dialysis (PD) or hemodialysis (HD)]; the study had a comparative design; patient outcomes (e.g. mortality, morbidity, peritonitis, switch to HD) were studied; volume was defined as a distinct number (e.g. continuous variable) or a cut-off value, or specialized/regionalized centers were analysed; the study did not describe a single center. All titles and abstracts were screened independently by two persons and the full texts of potentially eligible articles were then obtained and further assessed for eligibility against the review inclusion criteria. Any disagreements were resolved by discussion.

Data were extracted by one reviewer into structured summary tables and checked for accuracy by a second reviewer. Any disagreements were discussed until till consensus was reached. For each publication, we extracted data on patient characteristics; setting; data source(s); center volume definition; and results. In accordance with prior SRs investigating the volume–outcome relationship we extracted only the comparison for the highest volume category vs. the lowest volume category, as defined by the study authors. If necessary, effect measures such as odds ratios (OR), risk ratios (RR) or hazard ratios (HR) were recalculated in order to achieve that the results are always presented as comparing high volume centers with low volume centers (and not vice versa). Furthermore, we also calculated relevant effect measures if these were not reported by the study authors, but were available from the text. In case of unadjusted and adjusted analysis presented in the studies, we focused on the adjusted results. At all stages of data selection, data extraction and critical appraisal we contacted authors for clarification, if needed.

Methodological quality of the eligible studies was undertaken independently by two persons. Any disagreements were resolved by discussion. We utilized a quality assessment tool based on the Newcastle–Ottawa-Scale [19] that was recently used in a Cochrane review that investigated the volume–outcome relationship in colorectal cancer [20]. We made some minor adaptations when applying the tool to registry-based studies, around the last two questions dealing with incomplete data and missing data. It is quite likely that many registries might only incorporate data of cases with complete data. Under these circumstances a question on incomplete or missing data would be inappropriate as an assessment of quality. We made a considered judgement to replace these two questions for all registry-based studies, and evaluated the “quality of registry data” and the “selection of patients” instead. Both questions were previously used for a similar question related to the volume–outcome relationship in registry-based studies [12]. For all other studies, we used the original assessment tool from the Cochrane review by Archampong et al. [20], but omitted the first item concerning study design. For this particular clinical question, we deemed it inappropriate to include retrospective study design per se as a criterion for determining degree of bias. In the SR by Archampong et al., all registry-based studies were assessed to have a high risk of bias as a result of their retrospective design, despite the fact that many collect information prospectively. We obtained literature for additional information about the corresponding registries, if referenced by the study authors. Our modified assessment tool can be found in Additional file 2: Appendix S2, which has already been used successfully in a SR investigating the volume–outcome relationship in the Norwood procedure [21].

Because the identified studies were expected to be methodologically diverse (for example, different volume definitions), we decided a priori not to statistically combine results.

A p-level of <0.05 was considered statistically significant.

## Results

**Fig. 1:**
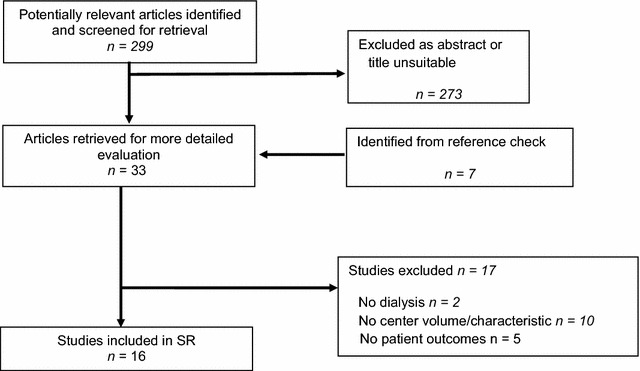
Flow chart

The search strategy generated 251 hits, of which 16 studies [22–37] met our inclusion criteria (Fig. 1). A list of excluded studies can be found in Additional file 3: Appendix S3. All 16 studies dealt with volume, while one study also contained data on hospital type [23]. In total, there were 15 registry-based studies and one clinical study [36]. Six studies were from the US [24, 25, 27, 34–36], four from France [26, 28, 31, 32], three from Canada [23, 29, 30], and each one study from Brazil [33], the Netherlands [22], and Romania [37]. All French studies were based on data from the French Peritoneal Dialysis Registry (RDPLF) and all Canadian studies were based on the Canadian Organ Replacement Register (CORR). The observation periods varied across all studies with the longest follow-up being 16 years in one study [23]. Similarly, the volume definitions varied extensively and were often not well reported. The number of volume categories ranged from two [22, 24, 25, 30, 36, 37] to seven [28].

### Study quality

Table 1 summarizes the results of the quality assessment. The study quality ranged from fair to good. There were only few items that were judged to have a high risk of bias. However, many items were judged to have an unclear risk of bias. This was mainly the result of insufficient reporting. In this context, many problems arose in particular when rating the outcome variables. However, it should be noted that an unclear risk of bias should be interpreted taking the character of the outcome variable under study into account. This explains the diverging outcome assessments for mortality and peritonitis, for example. Death as an outcome variable is more reliable than peritonitis. There was each one single best study for PD [28] and HD [27].

**Table 1:** Study quality

Study	Representativeness	Ascertainment	Comparability	Outcome			Registry quality/addressing incomplete data	Selection/missing data on primary interventions and outcomes
				Mortality/survival	Switch to HD/other treatment	Peritonitis		
Afolalu 2009 [25]	–	+	–	+	?	NA	?	–
Castrale 2010 [26]	–	?	+	+	?	?	+	+
Eisenstein 2008 [27]	+	+	+	+	NA	NA	–	+
Evans 2013 [28]	+	+	+	+	+	NA	+	+
Fenton 1997 [29]	+	+	+	+	NA	NA	?	?
Fenton 1993 [30]	–	?	?	+	NA	NA	?	–
Guo 2003 [24]	+	?	+	NA	?	NA	?	–
Huisman 2002 [22]	+	?	?	+	?	NA	+	+
Lobbedez 2011 [31]	–	+	+	NA	?	?	+	?
Lobbedez 2013 [32]	+	+	+	+	?	NA	+	–
Martin 2011 [33]	+	+	+	NA	NA	?	?	+
Mircescu 2014 [37]	+	?	+	+	NA	NA	?	?
Mujais 2006 [34]	?	?	?	NA	?	NA	?	?
Nolph 1986 [35]	–	?	?	NA	NA	?	+	?
Plantinga 2009 [36]	+	+	?	+	?	NA	?	?
Schaubel 2001 [23]	+	?	+	+	?	NA	?	?

+, fulfilled; ?, unclear; –, not fulfilled;* NA*, not applicable

### Center volume

#### PD

Ten studies analyzed PD technique survival by modeling switch to HD or any other treatment as an outcome [22–26, 28, 31, 32, 34, 36]. All studies showed a statistically significant result in favor of high volume facilities (Table 2). The relative effect measures ranged from 0.25 to 0.94 (median 0.73). However, it should be noted that four studies [26, 28, 31, 32] were based on the RDPLF and two [24, 34] studies were based on data from the Baxter Healthcare Corporation. Taking the observation periods into account, an overlap of participants will have occurred for both databases, although the overlap is likely to be greater for the RDPLF.

**Table 2:** **Results**

Author (pub year)	Study type	Region/country	Data source (period)	Dialysis	No. of patients	Volume definition	Hospital/centre volume [high/low]^c^	
Afolalu 2009 [25]	Registry	US (New England)	SIMS (2001–2005; follow-up NR)	PD	5003	Based on case volume in 12/2005	>25 vs. ≤25	1 year mortality^b^ OR = 1.06 (p = 0.53) 2 years mortality^b^ OR = 1.09 (p = 0.41) 1year switch to HDµ OR=0.73 (p=0.005) 2 years switch to HD^b^ OR = 0.74 (p = 0.03)
Castrale 2010 [26]	Registry	France	RDPLF (2000–2005; follow-up to 2007)	PD	1613	Number of all adult patients active on PD per day during study period	>30 vs. <20	Switch to HD HR = 0.56^a^ (0.37–0.86) Survival free of peritonitis HR = 1.01^a^ (0.77–1.33) Survival (study period) HR = 0.96^a^ (0.81–1.14)
Eisenstein 2008 [27]	Registry	US	USRDS (1996–1999; follow-up to 03/2001)	HD	1,86,596	NR	≥ 120 vs. ≤60	1 year survival^b^ OR = 1.24 (1.21–1.28) 2 years survival^b^ OR = 1.25 (1.22–1.29) 3 years survival^b^ OR = 1.29 (1.26–1.32) 4 years survival^b^ OR = 1.29 (1.26–1.32)
Evans 2013 [28]	Registry	France	RDPLF (2000–2009; follow-up to 2010)	PD	9602	12 month prior initiating treatment	>60 vs. ≤10	Switch to HD (5 years) HR = 0.46^a^ (0.26–0.81) Mortality (5 years) HR = 0.99^a^ (0.72–1.36) Transplantation (5 years) HR = 1.08^a^ (0.73–1.61)
Fenton 1997 [30]	Registry	Canada	CORR (1990–1994; follow-up to 1994)	HD/PD	10,633	Patients between 1981 and 1994	≥400 vs. <400	Mortality^b^ RR = 0.85^a^ (0.78–0.91)
Fenton 1993 [29]	Registry	Canada	CORR (1981–1991; follow-up to NR)	HD/PD	5476 (aged 65+)	NR	≥400 vs. <200	Mortality RR = 0.84^a^ (p = 0.068)
Guo 2003 [24]	Registry	USA	BHC (1999–2001; follow up to 02/2003)	PD	32,135	NR	>20 vs. <20	Switch to HD^b^ HR^a^ = 0.88 (p ≤ 0.0001)
Huismann 2002 [22]	Registry	The Netherlands	RENINE (1994–1999; follow up to 01/2000)	PD	4049	NR	≥20 vs. <20	Switch to HD RR^a^ = 0.60 (p < 0.0001) Mortality RR^a^ = NR (p > 0.05)
Lobbedez 2011 [31]	Registry	France	RDPLF (2002–2007; follow-up to 2008)	PD	4162	Mean number per day per centre over the study period	>30 vs. ≤20	Switch to HD HR^a^ = 0.72 (0.56–0.92) Peritonitis IRR^a^ = 0.88 (0.74–1.05) Enteric peritonitis IRR^a^ = 1.02 (0.73–1.43) Survival HR^a^ = 0.98 (0.80–1.20)
Lobbedez 2012 [32]	Registry	France	RDPLF (2002–2010; follow-up to 06/2011)	PD	9801	new patients per year	>20 vs. <10	Switch to HD HR^a^ = 0.83 (0.72–0.94) Renal recovery HR = 1.61 (1.02–2.55) Renal transplantation HR = 1.29 (1.13–1.48) Mortality HR = 1.11 (1.00–1.21)
Martin 2011 [33]	Registry	Brazil	BRAZPD (12/2004–10/2007; follow-up > 90 days)	PD	2032	Quintiles (over study period)	>156 vs. <40	Peritonitis HR^a^ = 0.67 (0.46–0.98)
Mircescu 2014 [37]	Registry	Romania	RRR (01/2008–12/2011; follow-up to 12/2012)	HD/PD	9252	Based on PD patients	≥20 vs. <20	Mortality HR^a^ = 0.88 (0.81–0.97)
Mujais 2006 [34]	Registry	US	BHC (2000–2003; follow-up to 06/2005)	PD	40,869	NR	>39 vs. <10	Survival HR = NR (p > 0.05) Switch to other treatment HR^a^ = 0.94 (p < 0.0001) [risk decrease for each census category higher] Transplantation HR^a^ = 1.17 (p < 0.0001) [risk decrease for each census category higher]
Nolph 1986 [35]	Registry	US	CAPD Registry (late 1981–12/1985; follow-up to 08/1985)	PD	6431	Center size defined by number of follow-up patients	>70 vs. <6	Peritonitis Median rate 1.13 vs. 1.25 (p = NR)
Plantinga 2009 [36]	Clinical	US	ESRD Quality (EQUAL) Study (10/1995–06/1998; follow-up NR)	PD	236	Number of dialyzed patients in October 1998	>50 vs. ≤50	Switch to HD HR^a^ = 0.25 (0.13–0.48) Cardiovascular events HR^a^ = 0.45 (0.29–0.70) Cardiovascular mortality HR^a^ = 0.63 (0.30–1.34) Mortality HR^a^ = 0.78 (0.48–1.26)
Schaubel 2001 [23]	Registry	Canada	CORR (1981–1997; follow-up to 1997)	PD	17,900	Cumulative number of patients treated	≥500 vs. ≤99	Mortality RR^a^ = 0.71 (0.63–0.81) Switch to HD RR^a^ = 0.83 (0.73–0.95)

*HD* hemodialysis, *PD* peritoneal dialysis, *IRR* incidence rate ratio, *RR* risk ration, *OR* odds ratio, *HR* hazard ratio, *SIMS* Standard Information Management System, *RDPLF* French Peritoneal Dialysis Registry, *USRDS* United States renal data system, *CORR* Canadian Organ Replacement Register, *BHC* Baxter Healthcare Corporation, *BRAZPD* Brazilian Peritoneal Dialysis Study, *RRR* Romanian Renal Registry, *CAPD* continuous ambulatory peritoneal dialysis, *RENINE* Dutch End-Stage Renal Disease Registry, *NR* not reported

^a^Adjusted analysis

^b^Re-analysed from data reported in the publication (not reported by the authors)

^c^Cut-off values presented for highest vs. lowest group

Nine studies analyzed patient survival by modeling mortality risk as an outcome [22, 23, 25, 26, 28, 31, 32, 34, 36]. Although the majority of studies indicated a slightly lower mortality or even a null effect for high volume facilities, only one study was able to prove this effect statistically yielding a RR = 0.71 (95 % CI 0.63–0.81) [23]. This analysis had an observation period from 1981 to 1997. Limiting the observation period from 1990 to 1997 resulted in a stronger effect with a RR = 0.51 (95 % CI 0.41–0.64).

Among four studies investigating peritonitis as an outcome [26, 31, 33, 35], one study based on data from the Brazilian Peritoneal Dialysis Study revealed lower rates of peritonitis in high volume centers yielding a HR = 0.67 (95 % CI 0.46–0.98) [33]. The other studies did not reach statistical significance.

#### HD

One large study found patients on HD treated in high volume facilities to survive longer [27]. The unadjusted OR for 1 year survival was 1.24 (95 % CI 1.21–1.28) when high volume facilities were compared to low volume facilities. The OR increased to 1.29 (95 % CI 1.26–1.32) for 4 year survival. Adjusted analyses were performed for patients with and without diabetes as their primary cause of end stage renal disease (ESRD). Statistical significant HRs were found in both groups per 10 unit increase.

#### PD and HD

Two studies analysed data from the CORR. One study found a statistically significant lower mortality (RR = 0.85; 95 % CI 0.78–0.91) in high volume hospitals [30]. The second study included only patients older than 64 years and resulted in a very similar effect yielding a RR = 0.84 (p = 0.07) but failed to reach statistical significance [29]. A third study for Romania found an adjusted HR of 0.88 (95 % CI 0.81–0.97) [37].

## Discussion

This article reviewed the existing literature on the volume–outcome relationship in dialysis. PD was studied most intensively. Therein, the majority of studies investigated either patient survival or technique survival as outcomes. With respect to PD, center volume has an effect on technique survival only. There was no effect on mortality risk or patient survival, while the evidence for peritonitis is inconclusive due to a limited number of studies. There is also not much evidence for HD, where only one study was available. Not withstanding, that study did show a strong effect of center volume on mortality. Studies analysing PD and HD together seem to support this finding. It can be concluded, that regarding mortality, there is no associated volume–outcome relationship in PD, but possibly in HD. No patient outcomes other than mortality have been studied for HD and HD/PD. Center type has only been investigated in one study, making it impossible to draw definitive conclusions.

### Limitations of the included studies

A number of methological issues impede consistent interpretation of the included studies. All studies are non-experimental raising questions in particular with respect to quality and completeness of data. Center volume as an explanatory variable can be confusing as the number of treated patients may classify the same center as either low volume or high volume, depending on geopolitical context. Center volume was defined in a number of different ways for all of our included studies. This is contrasts with surgical literature, where definitions of volume are more consistent yielding comparable results and providing convergent validity [38].

The time-varying nature of center volume as it pertains to dialysis is also problematic. For surgical procedures, patients undergo an operation and perhaps an inpatient stay depending on their requirements for recovery. Volume is modelled by annual caseloads, taking center volume either in the same year of patient’s admission [39] or 1 year before [40]. The temporal relationship between volume and outcome is therefore close. In contrast, ESRD is a chronic disease requiring indefinite treatment, with outcomes measured and reported over prolonged periods. Given the organic growth in ESRD, patient volumes are likely to vary and most likely increase over time in a given centre. If studies are performed over a prolonged period of observation, associations may be potentially biased by the use of baseline values for centre volume, or weakened if the authors take the mean over the whole study period.

In general, the authors of the included studies failed to justify their rationale for definition and categorization of centre volume. We cannot presume that their modelling choices were data driven, and many appear arbitrary. Interestingly, we found all studies analyzing volume as a categorical variable. There are advantages and disadvantages to this approach. Categorization of continuous data does avoid the presumption of any mathematical relationship, but can obscure associations and prevent deep understanding of data [41–43]. Prior research has shown that the volume–outcome relationship in surgery can be linear, stepwise, curvelinear or U-shaped, for example [44–47], and this requires further elucidation for dialysis.

As the included studies were observational, appropriate adjustment for possible confounders is critical. This is particularly relevant for studies investigating the volume–outcome relationship [48, 49]. All of the included studies made adjustments as possible, but were limited by data availability. A large but unquantifiable degree of residual confounding is therefore likely from omitted known patient-level and centre-level risk factors [15–18, 50–55].

### Limitations of the review

We acknowledge that our work has some limitations. First, we did not search for grey literature. This might have yielded additional information. However, we have cross-checked the references of all included studies and were able to include three studies that were not retrieved by our search strategy. Publication bias is difficult to assess. It should be noted that there are many dialysis registries available worldwide. Thus it is likely that more evidence could be produced within a short time from ongoing analysing at these registries.

Our systematic review did not aim to provide cut-off values. Cut-off values could be extended to minimum volume standards. Such standards already exist in surgery, but are well justified by robust volume–outcome data [56–60]. In dialysis, there are not only philosophical challenges as discussed, but also methodological issues to defining cut-off volumes that have not yet been explored in this area [61]. The contextual limitations of the included studies precluded us from defining or even suggesting cut-off values.

A point to note is that we did not use a validated tool to appraise study quality, but instead developed our own tool based on one from a previous Cochrane review. The rationale for this is that there is no tool that can be considered the ‘gold standard’ for this kind of clinical question/study designs. However, the quality assessment of a registry-based study remains challenging, resulting in numerous studies where the items could not be assessed as the publications provided no information on it. We have already faced this problem in an earlier SR [21]. Although there is much literature related to the quality of registries [62, 63], there is no accepted critical appraisal tool for registry-based studies or for registry-based studies specific items. The idea of a registry of registries has already been suggested [64], and this might be helpful for future SRs based on registry-based studies. Reporting standards for registry-based studies would further encourage authors to report relevant information to facilitate the assessment of the methodological quality.

This systematic review focused only on center volume or center type effects. As for many surgical procedures and other medical conditions, it is also possible to focus on the physician volume. One recent study found that nephrologists with the lowest patient mortality rates had significantly lower patient caseloads than nephrologists with the highest mortality rates [65].

## Conclusion

In conclusion, this systematic review supports a volume–outcome relationship in PD with respect to switch to HD or any other treatment. An effect on mortality is probably present in HD. Center volume itself can only be regarded as a proxy for quality of care, and further research is needed to identify the associations of center volume that are causally related to patient benefit. There is a surprising dearth of literature in the area, and such data would be valuable for quality initiatives in order to help identified low performing centers achieve better outcomes for their patients.

## Additional files

10.1186/s13104-015-1785-5 Search strategy.
10.1186/s13104-015-1785-5 Critical appraisal tool.
10.1186/s13104-015-1785-5 List of excluded studies.

## Abbreviations

RDPLF: French Peritoneal Dialysis Registry
HR: hazard ratio
HD: hemodialysis
OR: odds ratio
PD: peritoneal dialysis
RR: risk ratio
SR: systematic review
