# Supplementary material for: A systematic review of the impact of center volume in dialysis
Source: BMC Res Notes. 2015 Dec 22;8:812. doi: 10.1186/s13104-015-1785-5 (PMC4688925; doi:10.1186/s13104-015-1785-5)
Supplement: Supplementary file 1 — 10.1186/s13104-015-1785-5 Search strategy. [file 13104_2015_1785_MOESM1_ESM.docx]

**Appendix 1**

**Pubmed (10.11.2014)**

("Hospitals, High-Volume"[Mesh] OR "Hospitals, Low-Volume"[Mesh] OR regionali*[tiab] OR centreli*[tiab] OR centerli*[tiab] OR caseload[tiab] OR workload[tiab] OR “volume outcome”[tiab] OR “hospital volume”[tiab] OR “hospital volumes”[tiab] OR “hospital size”[tiab] OR “clinic size”[tiab] OR “clinic size”[tiab] OR “center volume”[tiab] OR “center volumes”[tiab] OR “center size”[tiab] OR “centre volume”[tiab] OR “centre size”[tiab] OR “patient volume”[tiab] OR “patient volumes”[tiab] OR “provider volumes”[tiab] OR “doctor volumes”[tiab] OR “procedure volume”[tiab] OR “procedure volumes”[tiab] OR “procedural volume”[tiab] OR “procedural volumes”[tiab] OR “facility volume”[tiab] OR “facility volumes”[tiab] OR “facility volume”[tiab] OR “treatment volume”[tiab] OR “treatment volumes”[tiab]) AND ("Dialysis"[Mesh] OR "Renal Dialysis"[Mesh] OR dialysis[tiab] OR hemodialysis[tiab] OR peritonealdialysis[tiab])

**Embase (10.11.2014)**

('high volume hospital'/exp OR 'low volume hospital'/exp OR regionali*:ti,ab OR centreli*:ti,ab OR centerli*:ti,ab OR caseload:ti,ab OR workload:ti,ab OR “volume outcome”:ti,ab OR “hospital volume”:ti,ab OR “hospital volumes”:ti,ab OR “hospital size”:ti,ab OR “clinic size”:ti,ab OR “clinic size”:ti,ab OR “center volume”:ti,ab OR “center volumes”:ti,ab OR “center size”:ti,ab OR “centre volume”:ti,ab OR “centre size”:ti,ab OR “patient volume”:ti,ab OR “patient volumes”:ti,ab OR “provider volumes”:ti,ab OR “doctor volumes”:ti,ab OR “procedure volume”:ti,ab OR “procedure volumes”:ti,ab OR “procedural volume”:ti,ab OR “procedural volumes”:ti,ab OR “facility volume”:ti,ab OR “facility volumes”:ti,ab OR “facility volume”:ti,ab OR “treatment volume”:ti,ab OR “treatment volumes”:ti,ab) AND ('dialysis'/exp OR 'renal replacement therapy'/exp OR dialysis:ti,ab OR hemodialysis:ti,ab OR peritonealdialysis:ti,ab)
