# Supplementary material for: A systematic review of the impact of center volume in dialysis
Source: BMC Res Notes. 2015 Dec 22;8:812. doi: 10.1186/s13104-015-1785-5 (PMC4688925; doi:10.1186/s13104-015-1785-5)
Supplement: Supplementary file 3 — 10.1186/s13104-015-1785-5 List of excluded studies. [file 13104_2015_1785_MOESM3_ESM.docx]

**Appendix 3**

**List of excluded studies**

**No dialysis**

Shlipak MG, Fried LF, Cushman M, Manolio TA, Peterson D, Stehman-Breen C, Bleyer A, Newman A, Siscovick D, Psaty B. Cardiovascular mortality risk in chronic kidney disease: comparison of traditional and novel risk factors. JAMA. 2005 Apr 13;293(14):1737-45.

Tangri, N., D. Ansell, and D. Naimark, Lack of a centre effect in UK renal units: Application of an artificial neural network model. Nephrology Dialysis Transplantation, 2006. 21(3): p. 743-748.

**No center volume/characteristic**

Chidambaram, M., et al., Patient and physician predictors of peritoneal dialysis technique failure: a population based, retrospective cohort study. Perit Dial Int, 2011. 31(5): p. 565-73.

De Cock, E., et al., Time savings associated with C.E.R.A. once monthly: A time-and-motion study in hemodialysis centers in five European countries. J Med Econ, 2013. 16(5): p. 648-656.

Elseviers, M., et al., European Practice Database: comparative results of the year 1 pilot project. EDTNA ERCA J, 2004. 30(2): p. 64-70.

Ghali, J.R., et al., Microbiology and outcomes of peritonitis in Australian peritoneal dialysis patients. Peritoneal Dialysis International, 2011. 31(6): p. 651-662.

Harley KT, Streja E, Rhee CM, Molnar MZ, Kovesdy CP, Amin AN, Kalantar-Zadeh K. Nephrologist caseload and hemodialysis patient survival in an urban cohort. J Am Soc Nephrol. 2013 Oct;24(10):1678-87. doi: 10.1681/ASN.2013020123. Epub 2013 Aug 8.

Kendrick, J. and I. Teitelbaum, Strategies for improving long-term survival in peritoneal dialysis patients. Clinical Journal of the American Society of Nephrology, 2010. 5(6): p. 1123-1131.

Li PK, Szeto CC. Success of the peritoneal dialysis programme in Hong Kong. Nephrol Dial Transplant. 2008 May;23(5):1475-8. doi: 10.1093/ndt/gfn068. Epub 2008 Feb 26.

Li, P.K.t. and K.M. Chow, Peritoneal Dialysis Patient Selection: Characteristics for Success. Adv Chronic Kidney Dis, 2009. 16(3): p. 160-168.

Stenvinkel P, Heimbürger O, Lindholm B. Wasting, but not malnutrition, predicts cardiovascular mortality in end-stage renal disease. Nephrol Dial Transplant. 2004 Sep;19(9):2181-3. Epub 2004 Jul 6.

Waikar, S.S., et al., Race and mortality after acute renal failure. Journal of the American Society of Nephrology, 2007. 18(10): p. 2740-2748.

**No patient outcomes**

Dong J, Han QF, Zhu TY, Ren YP, Chen JH, Zhao HP, Chen MH, Xu R, Wang Y, Hao CM, Zhang R, Zhang XH, Wang M, Tian N, Wang HY. The associations of uric acid, cardiovascular and all-cause mortality in peritoneal dialysis patients. PLoS One. 2014 Jan 8;9(1):e82342. doi: 10.1371/journal.pone.0082342. eCollection 2014.

Frankenfield DL, Sugarman JR, Presley RJ, Helgerson SD, Rocco MV. Impact of facility size and profit status on intermediate outcomes in chronic dialysis patients. Am J Kidney Dis. 2000 Aug;36(2):318-26.

Leonard, M.B., et al., Racial and center differences in hemodialysis adequacy in children treated at pediatric centers: a North American Pediatric Renal Transplant Cooperative Study (NAPRTCS) report. J Am Soc Nephrol, 2004. 15(11): p. 2923-32.

Obialo C, Zager PG, Myers OB, Hunt WC. Relationships of clinic size, geographic region, and race/ethnicity to the frequency of missed/shortened dialysis treatments. J Nephrol. 2014 Aug;27(4):425-30. doi: 10.1007/s40620-013-0035-y. Epub 2014 Jan 21.

Troidle, L., et al., Trends in chronic peritoneal dialysis utilization in New England. Adv Perit Dial, 2010. 26: p. 58-60.
